# Health workers’ strikes in low-income countries: the available evidence

**DOI:** 10.2471/BLT.18.225755

**Published:** 2019-05-14

**Authors:** Giuliano Russo, Lihui Xu, Michelle McIsaac, Marcelle Diane Matsika-Claquin, Ibadat Dhillon, Barbara McPake, James Campbell

**Affiliations:** a Centre for Primary Care and Public Health, Queen Mary University of London, 58 Turner Street, E1 2AB London, England.; b Health Workforce Department, World Health Organization, Geneva, Switzerland.; c Human Resource Directorate, Ministry of Health, Maputo, Mozambique.; d Nossal Institute for Global Health, Melbourne, Australia.

## Abstract

**Objective:**

To analyse the characteristics, frequency, drivers, outcomes and stakeholders of health workers’ strikes in low-income countries.

**Methods:**

We reviewed the published and grey literature from online sources for the years 2009 to 2018. We used four search strategies: (i) exploration of main health and social sciences databases; (ii) use of specialized websites on human resources for health and development; (iii) customized Google search; and (iv) consultation with experts to validate findings. To analyse individual strike episodes, pre-existing conditions and influencing actors, we developed a conceptual framework from the literature.

**Results:**

We identified 116 records reporting on 70 unique health workers’ strikes in 23 low-income countries during the period, accounting for 875 days of strike. Year 2018 had the highest number of events (17), corresponding to 170 work days lost. Strikes involving more than one professional category was the frequent strike modality (32 events), followed by strikes by physicians only (22 events). The most commonly reported cause was complaints about remuneration (63 events), followed by protest against the sector’s governance or policies (25 events) and safety of working conditions (10 events). Positive resolution was achieved more often when collective bargaining institutions and higher levels of government were involved in the negotiations.

**Conclusion:**

In low-income countries, some common features appear to exist in health sector strikes’ occurrence and actors involved in such events. Future research should focus on both individual events and regional patterns, to form an evidence base for mechanisms to prevent and resolve strikes.

## Résumé

**Objectif:**

Analyser les caractéristiques, la fréquence, les éléments moteurs, les conséquences et les acteurs des grèves des agents sanitaires dans des pays à faible revenu.

**Méthodes:**

Nous avons examiné la documentation publiée et grise provenant de sources en ligne pour les années 2009 à 2018. Nous avons utilisé quatre stratégies de recherche: (i) recherche dans les principales bases de données couvrant la santé et les sciences sociales; (ii) utilisation de sites Web spécialisés dans les ressources humaines pour la santé et le développement; (iii) recherches Google spécifiques; et (iv) consultation d'experts pour valider les conclusions. Pour analyser différents épisodes de grève, les conditions préexistantes et les acteurs influents, nous avons développé un cadre conceptuel à partir de la littérature.

**Résultats:**

Nous avons sélectionné 116 documents rendant compte de 70 grèves isolées des agents sanitaires dans 23 pays à faible revenu au cours de la période considérée, ce qui correspondait à 875 jours de grève. L'année 2018 présentait le plus grand nombre d'événements (17), soit 170 journées de travail perdues. Les grèves mobilisant plus d'une catégorie professionnelle étaient la modalité de grève la plus fréquente (32 événements), suivies par les grèves de médecins uniquement (22 événements). Les causes les plus communément avancées tenaient à des plaintes concernant la rémunération (63 événements), suivies par une contestation de la gouvernance ou des politiques du secteur (25 événements) et par la sécurité liée aux conditions de travail (10 événements). Les grèves ont plus souvent connu une issue positive lorsque des organismes de négociation collective et des ordres de gouvernement supérieurs participaient aux négociations.

**Conclusion:**

Certaines caractéristiques communes semblent exister dans les pays à faible revenu concernant la fréquence des grèves du secteur de la santé et les acteurs mobilisés lors de ces événements. Les futures recherches devront s'intéresser à la fois aux événements pris séparément et aux tendances régionales pour constituer un corpus de données relatif aux mécanismes de prévention et de résolution des grèves.****

## Resumen

**Objetivo:**

Analizar las características, la frecuencia, los motivos, los resultados y los participantes de las huelgas de los trabajadores sanitarios en los países de bajos ingresos.

**Métodos:**

Se revisó la literatura publicada y gris procedente de fuentes en línea desde el año 2009 hasta el 2018. Se utilizaron cuatro estrategias de búsqueda: (i) investigación de las principales bases de datos sobre salud y ciencias sociales; (ii) uso de páginas web especializadas en recursos humanos para la salud y el desarrollo; (iii) búsqueda personalizada en Google; y (iv) consulta con expertos para validar los resultados. Para analizar las huelgas individuales, las condiciones preexistentes y los actores que ejercen influencia, hemos desarrollado un marco conceptual a partir de la literatura.

**Resultados:**

Se identificaron 116 registros que informan acerca de 70 huelgas individuales de los trabajadores sanitarios en 23 países de bajos ingresos durante el periodo, lo que representa 875 días de huelga. En el año 2018 se concentró el mayor número de casos (17), que corresponden a 170 días de trabajo perdidos. Las huelgas que incluyen a más de una categoría de profesionales fueron la modalidad de huelga habitual (32 casos), seguidas de las huelgas hechas solo por médicos (22 casos). La causa más común fueron las quejas sobre la remuneración (63 casos), seguidas por las protestas contra la gestión o las políticas del sector (25 casos) y la seguridad de las condiciones laborales (10 casos). Se alcanzó una solución favorable en más ocasiones cuando las instituciones de negociación colectiva y las altas esferas del gobierno se involucraron en las negociaciones.

**Conclusión:**

En los países de ingresos bajos, parece que existen algunas características comunes en las huelgas del sector sanitario y en los participantes involucrados en dichos acontecimientos. Las próximas investigaciones se deberían centrar tanto en los casos individuales como en los patrones regionales, para crear una base empírica para que los mecanismos prevengan y resuelvan las huelgas.

## ملخص

**الغرض:**

تحليل خصائص إضرابات العاملين في القطاع الصحي، ومدى تكرارها، ودوافعها، ونتائجها، وأصحاب المصالح فيها، في البدلان ذات الدخل المنخفض.

**الطريقة:**

قمنا بمراجعة المؤلفات المنشورة وغير الرسمية من مصادر على الإنترنت خلال الأعوام 2009 إلى 2018. واتبعنا أربع استراتيجيات للبحث: (1) استكشاف قواعد البيانات الرئيسية للعلوم الصحية والاجتماعية؛ و(2) استخدام مواقع الإنترنت المتخصصة في الموارد البشرية للصحة والتنمية؛ و(3) البحث المتخصص على Google؛ و(4) التشاور مع الخبراء للتحقق من صحة النتائج. لتحليل أحداث الإضراب الفردية، والظروف الموجودة مسبقًا، والأطراف المؤثرة، قمنا بتطوير إطار عمل مفاهيمي من المؤلفات.

**النتائج:**

لقد عثرنا على 116 تسجيلاً عن 70 إضرابًا مختلفاً للعاملين في القطاع الصحي في 23 بلداً من أصحاب الدخل المنخفض، خلال هذه الفترة، وتمثل 875 يوما من الإضراب. شهد عام 2018 أعلى رقم من الأحداث (17)، وهو ما يعادل 170 يوم عمل ضائع. الإضرابات التي تشمل أكثر من فئة مهنية واحدة كانت وسيلة الإضراب المتكررة (32 حدثاً)، تليها الإضرابات بواسطة الأطباء فقط (22 حدثا). كان السبب المعلن الأكثر شيوعًا هو الشكاوى المتعلقة بالأجور (63 حدثًا)، تليها الاحتجاج على حوكمة القطاع أو سياساته (25 حدثًا)، وظروف السلامة في العمل (10 أحداث). وكان يتم تحقيق قرار إيجابي في أغلب الأحوال عندما تشارك مؤسسات للمفاوضة الجماعية مع مستويات أعلى من التمثيل الحكومي في المفاوضات.

**الاستنتاج:**

يبدو في البلدان منخفضة الدخل وجود بعض الخصائص الشائعة في معدل حدوث اضرابات القطاع الصحي، والأطراف المشاركة في هذه الأحداث. يجب أن تركز الأبحاث المستقبلية على كل من الأحداث الفردية والأنماط الإقليمية، حتى تشكل قاعدة أدلة للآليات لمنع حدوث الإضرابات، فضلاً عن حلها.

## 摘要

**目的:**

旨在分析低收入国家医疗保健工作者罢工的特征、频率、驱动因素、结果及利益相关者。

**方法:**

我们对 2009 年至 2018 年的在线资源进行了回顾，包括已发表文献和灰色文献。我们采用四种搜索策略：(i) 探索主要的医疗保健和社会科学数据库；(ii) 使用医疗保健与发展的人力资源专门网站；(iii) 定制谷歌搜索；(iv) 与专家磋商以验证结果。旨在分析个别罢工事件、既存问题和具有影响力的参与者，我们从文献中提出了一个概念框架。

**结果:**

我们确定了 116 份记录，报告了在此期间 23 个低收入国家 70 个医疗保健工作者的特殊罢工事件，占罢工总天数的 875 天。2018 年发生的罢工事件数量最多（为 17），相当于损失了 170 个工作日。频繁罢工（32 个事件）是包含不止一类的罢工，其次是仅有医生的罢工（22 个事件）。报告的最常见原因是薪酬投诉（63 个事件），其次是抗议行业治理或政策（25 个事件）和工作场所安全（10 个事件）。当集体谈判机构和更高级别的政府机构参与谈判时，更易取得积极的解决方案。

**结论:**

在低收入国家中，医疗保健部门罢工事件以及此类事件的参与者似乎存在一些共同特征。未来的研究应关注个体事件和区域模式，以形成预防和解决罢工机制的证据基础。****

## Резюме

**Цель:**

Проанализировать характеристики, частоту, побудительные причины, результаты и участников забастовок медицинских работников в странах с низким уровнем доходов.

**Методы:**

Авторы изучили опубликованную и неиндексированную литературу из сетевых источников за период с 2009 по 2018 год. Были использованы четыре стратегии поиска: (i) изучение основных баз данных здравоохранения и социальных наук; (ii) использование специализированных кадровых веб-сайтов в сфере здравоохранения и развития; (iii) специализированный поиск в Google; (iv) консультации со специалистами для валидации сделанных выводов. Для анализа отдельных эпизодов забастовок, предшествовавших условий и влияющих на ситуацию действующих лиц была разработана концептуальная схема по данным литературы.

**Результаты:**

Авторы обнаружили 116 документов, в которых сообщалось о 70 не связанных между собой забастовках медицинских работников в 23 странах с низким уровнем дохода; общая продолжительность забастовок в указанный период составила 875 дней. Больше всего таких событий приходилось на 2018 год (17), в общей сложности потери рабочего времени составили 170 дней. Забастовки, распространявшиеся на работников более чем одной профессиональной категории, были частыми (32 случая), за ними шли забастовки только врачей (22 случая). Чаще всего причинами забастовки являлись: недовольство уровнем заработной платы (63 случая), недовольство управлением сектором или принимаемыми стратегиями (25 случаев) и безопасность условий труда (10 случаев). Положительный результат чаще достигался, когда в переговорах участвовали коллективные переговорные институты и когда в процесс были вовлечены высокопоставленные правительственные чиновники.

**Вывод:**

Для стран с низким уровнем дохода характерны некоторые общие черты, касающиеся возникновения забастовок в секторе здравоохранения и сторон, участвующих в них. Впредь следует сосредоточить усилия исследователей как на индивидуальных вмешательствах, так и на общности региональных черт, чтобы сформировать базу доказательств для механизмов предотвращения и разрешения случаев забастовок.

## Introduction

Workers’ strikes or industrial action are identified as “…the collective withholding of labour/services by a category of professionals, for the purpose of extracting concessions or benefits, typically for the economic benefits of the strikers.”^1^ The right to strike is widely considered a civil right and is often part of a country’s legal system.^2^ However, for some professional groups, including health workers, strikes might have implications beyond the involved parties. Health workers’ strikes has been purported as putting patients at risk of serious harm and potentially contradict health workers’ duties to care for their patients^1^^,^^3^ and evidence from high-income settings shows that nurses’ strikes can affect hospital mortality.^4^ Although doctors’ strikes in high-income settings may not necessarily increase patients’ mortality in the short term,^5^ they can severely disrupt the provision of health-care services, with significant political, organizational and financial implications.^6^ In middle-income countries, some evidence shows that physician strikes can lead to a decrease in clinical activities and increase in mortality.^7^^,^^8^ Some have argued that such strikes would be justified if directed towards improving workers’ conditions and their ability to care for future patients^9^ and that doctors’ strikes may be morally acceptable if proportionate and properly communicated.^10^

Health workers’ strikes are of growing concerns to the international health community and organizations aiming to ensure health and access to health services for all. Health workers play a central role in achieving universal health coverage (UHC), and interruptions in health services not only hold implications for UHC, but suggest unresolved labour and governance issues in health sectors, particularly in some lower-income settings with poor governance and regulations.

Determinants of health workers’ strikes can be diverse. For example, gross domestic product (GDP) growth and widening wage differentials across professions have been associated with industrial action in the private sector in the United States of America.^11^ Compensation differentials appear to be a central determinant of public sector strikes, as wage disputes are informed by comparisons with private sector salaries, or with public sector salaries of or similar-level professions.^12^ Economic theorists suggest that the likelihood of strike action increases when a country’s general economic conditions improve and unemployment rates are low,^11^ because renegotiation on how to share the society’s increased wealth among its members is needed. Improving the profession’s social and political position within the society can be another motivation for strikes.^13^

Currently, the main mechanisms for strike resolution in most high-income countries are outright prohibition of strikes for certain public sectors, formal systems for impasse procedures and forms of binding arbitration between the negotiating parties.^12^^,^^14^ In the public sector, formal systems of impasse procedures, such as conventional, binding and final offer arbitration, are often in place, where governments assist employers and unions to help resolve disputes.^12^

Medical associations and councils play a critical role in not only triggering, but also mediating strikes because of their role of unions and self-regulatory bodies.^15^ For example, in Mozambique and the United Republic of Tanzania, medical associations were formed to negotiate remuneration grievances from junior doctors, in contrast to the official medical councils representing the interests of more senior cadres (Arroz J, *Associação Médica de Moçambique*, unpublished data, 2014).^16^

In low-income countries, data on health workers’ strikes are scarce and the implications of such strikes are potentially wide reaching when health systems are fragile. This paper analyses the evidence on health sector strikes in low-income countries in the last decade with the aim of understanding their characteristics and drivers and providing a baseline for future research. 

## Methods

### Search strategy

We adapted a published method^17^ to systematically search the grey literature, focusing primarily on online resources given that health workers’ strike episodes are more likely to be reported by the media than in academic publications. For this work, we used the Luxembourg’s definition of grey literature, covering documentation produced by all levels of government, academics, business and industry in print and electronic formats, produced by non-commercial publishers.^18^

First, we searched general health and social science databases (PubMed®, Scopus, EconLit and Web of Science) between July and August 2018 using the following search terms: “Country name” AND “strike” OR “industrial action” AND “physicians” OR “doctors” OR “nurses” OR “pharmacists” OR “dentists” OR “midwives” OR “health worker” OR “hospitals.”

We then searched specialist development databases (ReliefWeb, World Health Organization’s (WHO’s) Index Medicus and Global Nonviolent Action Database) and dedicated websites on labour and human resources for health (Public Services International, ILO, WHO and the World Bank) between July and September 2018 using combinations of the above search terms. 

We then conducted customized Google searches (Box 1) of media reports in English and if we did not identify any record in English, we searched in one of the official languages of the countries reviewed (Spanish, French and Portuguese). The search covered international websites of BBC News Africa, Al Jazeera, Fox News, France 24 Observers, Thomson Reuters Foundation News, Reuters and the local news networks Mail & Guardian, AllAfrica, MedAfrica Times, Medical Xpress, Guinee Matin, Caribbean Life News, Africanews, as well as on national Medical Associations news databases, using combinations of the above search words and their correspondent in the local language. For identified records in local languages spoken by at least a million people in one or more of the low-income countries (Amharic, Pashtun and Swahili), we used Google Translator. The customized searches were updated in March 2019 (number of Google hits are available from the corresponding author).

Box 1 **Customized Google searches for health worker’s strikes in low-income countries**

We searched information for each of the 31 low-income countries using the search string in English: “[country name] strikes physician” OR “doctor” OR “nurse” OR “pharmacist” OR “dentist” OR “midwife” OR “health worker” OR “hospital.”

For countries with French as official languages, we used a search string in French if we did not identify any records using the English search string: “[Nom du pays] grève médecin” OR “infirmier” OR “pharmacien” OR “dentiste” OR “sages-femme” OR “agent de santé” OR “hôpital”

For countries with Spanish as official languages, we used a search string in Spanish if we did not identify any records using the English search string: “[Nombre del país] and huelga” OR “medico” or “enfermera/o” OR “farmaceutico” OR “dentista” OR “comadrona” OR “and trabajadores del sector salud” OR “hospital”

For countries with Portuguese as official languages, we used a search string in Portuguese if we did not identify any records using the English search string: “[Nome do país] and greve” OR “doutor” OR “enfermeiras” OR “farmacêutico” OR “dentista” OR “parterira” OR “trabalhadores do sector saúde” OR “hospital”

Finally, for countries which we did not obtain any information on health workers’ strikes, one of the authors consulted nine local and international health sector experts in June and August 2018. To improve the comprehensiveness of this strategy, we chose content experts to reflect a diversity of disciplines and geographical areas relevant for the strike events (list of experts available from the corresponding author).

### Inclusion and exclusion criteria

We used the World Bank’s 2017 classification to identify the 31 low-income countries for our search.^19^ Titles were included if they reported on “a temporary work stoppage effected by one or more groups of workers with a view of enforcing or resisting demands or expressing grievances or supporting other workers in their demands or grievances.”^20^ We included reports of events that took place between January 2009 and December 2018.

Information on strikes based on only social media reports were excluded due to the inability to triangulate and validate the information. Titles on alternative forms of industrial action were not included, such as go-slow strikes, threats to strike or silent protest marches. Given the inclusion of macroeconomic factors and a need to ensure consistency across health-care settings, only nation-wide or province-wide strikes were considered. Strike episodes that were highly localized (e.g. in a single hospital) and reports on threats of strike action were excluded.

### Framework

**Fig. 1:**
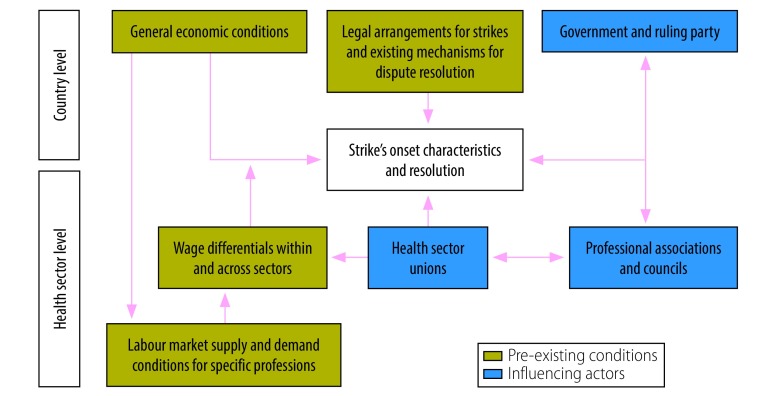
Conceptual framework for health sector strikes in low-income countries

To guide the data extraction and the analysis of the individual strike events identified through our search, we developed a framework that summarizes the concepts from the economic, political economy and health system research literature on health sector strikes, and helps to understand the linkages between pre-existing conditions, relevant influencing actors and their interaction (Fig. 1).

The framework highlights the need to consider the micro as well as macro dimensions of health sector strikes. For example, pre-existing economic and legal conditions, including economic growth, wage and unemployment levels, or the existence of mechanisms for resolution of disputes, are associated with strike onsets. Actors, such as unions, government, parties and professional associations, play a role in driving and resolving the disputes.

The approach to develop this framework was in line with the High-Level Commission on Health Employment and Economic Growth,^21^ which highlights the interaction between health workers, the health sector and the macroeconomic context of a country. The framework also draws from the method used by the Organisation for Economic Cooperation and Development (OECD) and International Labour Organization (ILO) to collect data on strikes and collective bargaining systems, and to analyse the impact of such events on labour markets in high-income countries.^22^

### Data extraction

We created two Excel spreadsheets with the pivot table feature (Microsoft, Redmond, United States of America) to store and analyse the information from records included in the study.

All identified records were screened by two authors. From records meeting the inclusion criteria, they extracted data on the length of strike episodes, main actors involved, relevant strike features and resolutions of events, following the conceptual framework. For validation, we triangulated the information on unique strike events identified from the eligible records. We regarded international news sources as more trustworthy than national and local ones. When two sources gave conflicting accounts of causes and resolution, we sought for a further source of information for clarification. 

We extracted GDP per capita (in United States dollars) and GDP growth data for the years 2009 to 2016 from the World Development Indicators database.^19^ For the years 2017 and 2018, we obtained current GDP per capita and country GDP growth from the International Monetary Fund.^23^ We retrieved information on unemployment rates from the ILOSTAT database^24^ for the years 2009 to 2017. Unemployment rates for 2018 were either incomplete or not available by March 2019, we therefore assumed they were unchanged from 2017.

We followed the Preferred Reporting Items for Systematic Reviews and Meta-Analyses guidelines^25^ to report on the review.

## Results

**Fig. 2:**
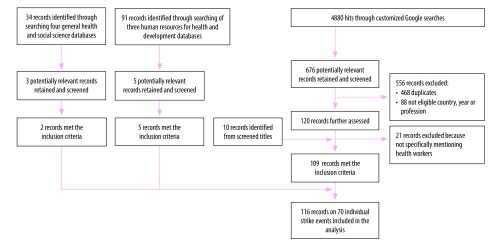
Flowchart of the included records on health workers’ strikes in low-income countries

The review of the general health and social science databases yielded an initial 34 titles from the published literature, three of which we retained after screening. Searching specialist databases resulted in 91 potentially relevant titles, of which five reports on individual health workers’ strikes were eligible for inclusion. We identified an initial 676 records, of which 109 met the inclusion criteria after elimination of duplicates (available from corresponding author) when doing the customized Google searches. In total, 116 reports covering 70 unique strike episodes in low-income countries met our inclusion criteria. Of the reports identified, most (103) were online media reports, five human resources for health reports from ReliefWeb and The World Bank databases and two academic publications (Fig. 2).^16^^,^^26^

We identified strike episodes across 23 low-income countries between 2009 and 2018 (Table 1; available at: http://www.who.int/bulletin/volumes/96/7/18-225755). Eight low-income countries had no report of health workers’ strikes during this period (Afghanistan, Central Africa Republic, Eritrea, Ethiopia, Democratic People's Republic of Korea, Guinea, Myanmar and Rwanda). The experts identified six initial records from these countries, however, none of these records were eligible. 

**Table 1:** Characteristics of strike episodes in 23 low-income countries, 2009–2018

Country	Month and year	Duration, days	Type of worker involved	Motive	Actors involved	Outcome and resolution
Benin	May 2009	ND	All health workers	ND	ND	ND
	Oct 2014	ND	All health workers	To dispute delayed remuneration and wage differences between paramedical and doctors	*Syndicat du personnel de l’Homel*	ND
	Sep 2017	60	All health workers	To dispute pay cut and government’s health reform plan and to demand better working conditions	Benin health sector unions and Ministry of Development	About 3 million United States dollars would be made available towards improving salaries and working conditions
	Jan 2018	30	All health workers	To dispute government’s health and education reform plan	Seven trade unions representing public sector workers in areas, such as health and the justice system	ND
Burkina Faso	Nov 2012	4	All health workers	ND	*Syndicat des travailleurs de la santé humaine et animale* versus the health ministry	ND
	Apr 2013	4	All health workers	Remuneration and better working conditions. To dispute a lawsuit against a health worker who caused a patient’s death during a strike in 2012	*Syndicat des travailleurs de la santé humaine et animale* versus the health ministry	ND
	Feb 2015	2	Workers from several sectors	To demand a major price reduction of gasoline and justice for crimes committed during the past regimes	ND	ND
	Nov 2016	3	All health workers	To demand for a pay rise and increased allowances	*Syndicat des travailleurs de la santé humaine et animale* versus the health ministry	In March 2017, a memorandum of understanding signed a new construction plan for hospital infrastructures and measures addressing national generic drugs crisis
	Jan 2018	1	All health workers	To urge for the implementation of an agreement reached on March 2017. To dispute a lawsuit against a health worker who caused a patient’s death during a strike in 2012	*Syndicat des travailleurs de la santé humaine et animale*, *Syndicat autonome des infirmièrs et infirmières du Burkina*, *Syndicat des sages-femmes et accoucheuses du Burkina Faso, Syndicat des médecins du Burkina*, *Syndicat des pharmaciens du Burkina Faso* and *Syndicat des travailleurs de l’administration hospitalière et des services de Santé*^a^ versus Prime Minister's office and the health ministry	In Feb 2018, a memorandum of understanding was signed with a plan to increase the overall remuneration package for public sector health workers
Burundi	May 2009	60	All health workers	To demand for a pay rise. To urge implementation of agreement	ND	ND
Chad	Oct 2016	ND	Workers from several sectors	To dispute delayed payment. To dispute government’s measures addressing public finance issues	*Union des Syndicats du Tchad* and several independent unions	Three months later, government confirmed its intention to amend the law on striking and industrial action. The changes included propositions whereby public servants are no longer paid on non-working days
	Feb 2018	ND	Workers from several sectors	To dispute a unilateral pay cut	Public Services International sent a letter to the President denouncing cuts to health-care services and calling for workers to be viewed as key actors in the realization of the country's development.	ND
Comoros	Nov 2009	45	All health workers	To demand for a pay rise. To dispute delayed payment and the President's resignation	ND	ND
Democratic Republic of the Congo	Sep 2013	ND	Doctors	ND	*Syndicat national des médecins du Congo*	ND
	Jun 2017	5	Doctors	To oppose the lack of security following the murder of two doctors	The provincial Medical Board: *l’Ordre des médecins du Nord-Kivu*	ND
	Aug 2017	15	Doctors	To demand for a pay rise in a context of high inflation rate and to dispute delayed payment	*Syndicat national des médecins du Congo* versus the Prime minister's Office	Government promised to include doctor’s salary increase in its 2018 budget
	Apr 2018	ND	Doctors	Remuneration and to urge the government to implement the agreement signed in 2017	*Syndicat national des médecins du Congo versus* Prime minister's Office	Government made a proposal, but no agreement reached for the time being
Gambia	Mar 2018	30	Doctors	In reaction to allegations by the Health Minister that medical practitioners steal drugs from public hospitals to stock their own private clinics	Gambia Association of Resident Doctors, and Vice President’s Office versus the health ministry	Establishment of a taskforce to investigate the case and oversee reforms in the health sector
Guinea-Bissau	Jul 2011	5	All health workers	To demand back payment of night shift bonus and better working conditions. To urge the government to hire medical interns who have completed their training	ND	ND
	Apr 2016	ND	Workers from several sectors	To demand payment of salary in arrears and allowances	ND	ND
Haiti	Apr 2016	150	Medical graduates and junior doctors	To dispute delayed payment, unsafe working places and shortage of basic medical supplies. Calling for health sector reforms	Health ministry	Pay demands met and conditions improved, including a gradual pay adjustment for residents
	Jan 2017	ND	All health workers	To demand for pay rises, fair salary adjustment for all professional categories and better working conditions. To dispute wage differences between nurses, non-medical personnel and doctors	*Syndicat des travailleurs de la santé de l’Hôpital de l’Université d’État d’Haïti* versus the health ministry	ND
Liberia	Jul 2013	10	All health workers	To dispute delayed payments; to demand for a pay rise, better working conditions and full-time employment status to long-time contracted workers	National Health Workers' Association of Liberia versus the health ministry	Negotiations taken place
	Oct 2014	1	All health workers	To demand danger pay and medical equipment for Ebola care	Liberian Health Workers Association	ND
	May 2018	5	Doctors	Arrears owed to interns doctors, low pay of medical doctors and poor working condition	Liberia Medical and Dental Association	Doctors suspended strike after government’s commitment to resolution
Madagascar	Jan 2010	30	Doctors	To demand for a pay rise, including a revision of payment scale and allowances. To dispute wage differences between doctors and military officers	*Syndicat des fonctionnaires* versus health ministry	ND
	Jun 2012	ND	All health workers	To urge the government to implement its 2010 commitment for salary increases and better working conditions	*Syndicat des infirmiers et sages-femmes de Madagascar*, *syndicat des médecins fonctionnaires de Madagascar*	ND
	Jul 2012	30	Workers from several sectors	To demand a pay rise and better working conditions	Health ministry	Salary suspension for strike leaders (paramedics) and arrests of doctors
Malawi	Mar 2015	ND	All health workers	To dispute wage differences between health workers and other public sector workers	Christian Health Association of Malawi	ND
Mali	Feb 2014	2	All health workers	To express dissatisfaction over the change of a union official	*Syndicat national de la santé, de l’action sociale et de la promotion de la famille*, *Syndicat autonome des cadres medicaux* and *Syndicat national des medecins du Mali*	ND
	Mar 2017	30	Workers from several sectors	To urge for an immediate implementation of a memorandum of understanding in 2016 and to demand for regularization of contracted personnel	National Union of Health, Social Action and Family Promotion versus Ministry of Commerce	Agreement reached
Mozambique	Jan 2013	10	Doctors	To demand better remuneration and working conditions	Association of Mozambican Medics versus the health ministry and public administration ministry	Military doctors filled the service gap. Memorandum of understanding signed in Jan 2013, with more general public sector wage settlement taken into effect in April 2013
	May 2013	10	Doctors	To demand for a pay rise of 100%	Association of Mozambican Medics	Negotiations taken place
	Jun 2013	27	All health workers	To demand for a pay rise and better working conditions. To dispute wage differences between health workers and judiciary officers	Association of Mozambican Medics versus the Prime Minister's Office and the health ministry	Military doctors, interns and Red Cross volunteers filled the service gap. Strike ended without reaching an agreement
Nepal	Jan 2014	6	Doctors	To demand the removal of the government-appointed head of Tribhuwan University Teaching Hospital	Nepal Medical Association	The executive removed; no further political interference in the medical education system. Establishment of a committee to transform the government institute to an independent university
	Apr 2015	1	Doctors	To demand reforms in medical education and health-care services	Nepal Medical Association	ND
	Sep 2017	8	All health workers	To dispute a Cabinet decision on health worker’s liability when patients die after treatment	Nepal Medical Association versus the health ministry and the Prime Minister's Office	Agreement reached between the striking parts facilitated by the office of the Prime Minister
Niger	Nov 2011	ND	All health workers	To demand better remuneration and working conditions	*Syndicat Unique de ta Santé et de l‘Action Sociale* versus the health ministry	Memorandum of understanding signed in 27 Dec 2011
	May 2013	ND	All health workers	To protest over drastically reduced allowances	Health ministry	ND
	Apr 2014	2	Physicians specialists	To dispute delayed payment and wage differences between public specialist doctors and civil servants	*Syndicat des médecins spécialistes du Niger *	ND
	Nov 2015	5	Specialists	To urge the government to implement its 2011 commitment on special salary and payment increases for specialist doctors. To dispute wage differences between public specialist doctors and civil servants	*Syndicat des mle syndicat des médecins spécialistes du Niger* versus the health ministry	ND
	Nov 2016	5	Specialists	To urge the government to implement its 2011 commitment on special salary and payment increases for specialist doctors. Part of recurrent strikes in since 2014	*Syndicat des mle syndicat des médecins spécialistes du Niger* versus the health ministry	ND
	Nov 2017	5	Specialists	To demand for better remuneration/ payment valuing specialist doctors' extra 5-years study and efforts, and better working conditions. To dispute wage differences between public specialist doctors and civil servants.Part of recurrent strikes in since 2014	*Syndicat des mle syndicat des médecins spécialistes du Niger* versus the health ministry	ND
	Jan 2018	1	All health workers	To dispute delayed payment	*Syndicat national des agents contractuels de santé de terrain*	ND
Senegal	Jun 2015	2	Doctors	To urge the implementation of a protocol agreement. To demand the right for health workers to be reinstated in their jobs	Autonomous Union of Doctors of Senegal	ND
	Sep 2016	2	Doctors	To express dissatisfaction over the government, while asking for dialogue and negotiation. To demand for career promotions and tenure; immediate appointment of health workers for the medical commission of the pilgrimage to Mecca. To dispute the suspension of the supply of electricity and water sanitary structures	Autonomous Union of Doctors of Senegal	ND
	Mar 2018	3	Doctors	To reiterate the 2014 demands for system of allowances, effectiveness of equipment loans, social housing, valuation of the work of regional doctors and raising doctors’ retirement age to 65 years	Autonomous Union of Doctors of Senegal and National Union of Health Workers versus the Prime Minister's Office	Concrete proposals from the government and a follow-up meeting to monitor the implementation
	Apr 2018	ND	All health workers	Similar demand as doctors made in Mar 2018	Single Union of Health Workers, National Union of Health Workers, *Syndicat Autonome des Agents de la Santé* versus Prime Minister’s Office	ND
	Sep 2018	3	Medical graduates and junior doctors	To demand for improving the status of interns, recruitment of interns in the public service; compliance of health ministry with the medical care law, payment of specialization costs	*Association des medecins internes du Senegal* versus *Ministere de la Santé et de l'Action sociale*	ND
Sierra Leone	Mar 2010	10	All health workers	To demand for a pay rise	President's office	The President agreed to increase doctors' salaries; unclear if nurses' salaries were increased
	Sep 2014	ND	All health workers	To demand better remuneration and working conditions	ND	ND
	Nov 2014	ND	All health workers	To dispute government's failure to pay an agreed weekly hazard payment	ND	ND
	Dec 2014	1	Medical graduates and junior doctors	To protest over inadequate equipment to fight the Ebola outbreak	Junior Doctors Association of Freetown's Connaught Hospital	ND
	Sep 2017	1	Multiple types of health workers	To dispute the health ministry and Sanitation’s refusal to sign the Community Health Practitioners Act 2017. The act is mainly about community health worker's status	Sierra Leone Association of Community Health Workers	ND
	Dec 2018	13	Doctors	To demand for pay rises, medical equipment and health insurance for medical professionals	Sierra Leone Medical and Dental Association and the Junior Doctors' Association versus Multiple Ministries (Labour, Finance and Health)	Government met the key demands of health workers
South Sudan	Mar 2013	ND	All health workers	To demand bonuses when oil production in the country resumed in early Mar 2013	Health ministry	ND
	Sep 2014	1	All health workers	To dispute delayed payment. To demand for a pay rise and better working hours and shift arrangements	Health Workers' Union versus the health ministry; the President's Office	Health ministry and the President's Office intervened and promised to solve the issues
Togo	Jun 2011	4	Doctors	To urge the government to respect its commitments made in 2016 regarding work allowances	*Syndicat National des Praticiens Hospitaliers du Togo*^a^	ND
	Jan 2018	2	Workers from several sectors	To demand for better equipment and more nursing staff as a part of an opposition parties coalition-led movement against the government and current president	*Syndicat National des Praticiens Hospitaliers du Togo*^a^	ND
	Mar 2018	4	Doctors	To demand better working conditions and long-term appointments for contract workers, recruitment of staff in public health training as well as reinstatement of several staff who were wrongly dismissed from work	*Syndicat National des Praticiens Hospitaliers du Togo*^a^	No agreement and solutions reached
	Apr 2018	3	Public sector workers	A follow-up on the 2018 series of strikes	*Syndicat National des Praticiens Hospitaliers du Togo*	ND
Uganda	Nov 2017	20	Doctors	To dispute low salaries and shortages of essential supplies	Uganda Medical Association; Uganda Nurses and Midwives Union versus the health ministry	Government committed to allowances for physicians, emergency supplies and entry level salary increase for doctors
United Republic of Tanzania	Jan 2012	60	All health workers	To demand for a pay rise and that more equipment and medicines are available in hospitals. To dispute the leadership of the health ministry	Medical Association of the United Republic of Tanzania versus the health ministry and President's office	President's intervened, because previous talks with senior government officials, including the Prime Minister, ended in stalemate
	Jun 2012	ND	Doctors	To demand for a pay rise. To support a medical group leader who claimed that he was kidnapped and tortured by strangers	Medical Association of the United Republic of Tanzania versus President's office	ND
Zimbabwe	Oct 2014	20	Medical graduates and junior doctors	To demand for better salaries and working conditions	Zimbabwe Hospital Doctors Association versus the health ministry	Agreement reached between the parts to terminate the strike
	Mar 2016	30	Medical graduates and junior doctors	To dispute the Health Service Board over a recent decision to employ over 60 doctors as contract workers, with unfair revenue package	Government’s Health Services Board	Junior doctors signed contracts
	Feb 2017	21	All health workers	To demand better remuneration	Zimbabwe Hospital Doctors Association versus Ministry of Health and Child Care and Health Services Board	Army medics filled the service gap. The government agreed to improve the remuneration with immediate effect
	Mar 2018	30	Medical graduates and junior doctors	To demand better remuneration and working conditions	Zimbabwe Hospital Doctors Association versus Ministry of Health and Child Care and Health Services Board	A pay deal was reached after the intervention of the President
	Apr 2018	5	Nurses	To demand better remuneration and working conditions	Zimbabwe Nurses Association versus the Vice President’s Office and health ministry	Unemployed or retired nurses filled the service gap. The government sacked more than 10 000 nurses who went on strike; nurses resumed work and began negotiations with the authorities
	Dec 2018	40	Medical graduates and junior doctors	To demand increase in monthly salaries and on-call pay, and for the government to address the shortage of medical supplies and equipment in hospitals	Zimbabwe Hospital Doctors Association versus Ministry of Health and Child Care	ND

ND: not detected.

^a^ Public Services International members.

All included health workers’ strikes were suspension of service provision, with only emergency services guaranteed in hospitals’ emergency and resuscitation departments.

### Frequency and duration

**Fig. 3:**
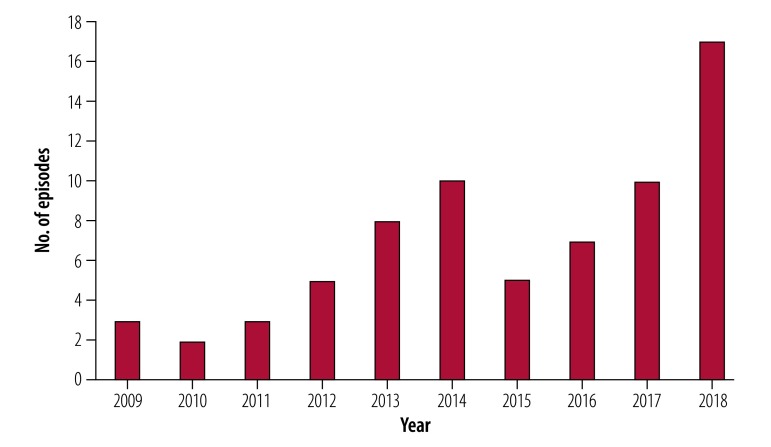
Health workers’ strikes across 23 low-income countries, 2009–2018

The median number of strike events was six per year, however, the data collected show an irregular pattern of episodes over the decade, with most strikes (49 events) recorded in the last five years. The years 2014 and 2018 registered the highest number of episodes, 10 and 17 events, respectively (Fig. 3). The year 2018 had the highest number of total work days lost (170), while Niger recorded the largest number of reported strikes (seven events), followed by Sierra Leone and Zimbabwe (six events; Table 1).

From the records reporting on number of days of health workers’ strikes, we calculated that a total of 875 working days were lost between 2009 and 2018, with a median number of 77.5 working days lost per year. That is, on every third working day on average, there was a strike taking place in the health sector in a low-income country during this period. Strike episodes lasted an average of 12.5 days, although some strikes protracted for months, such as the general health sector strikes in Haiti in 2016. Some strikes were recurring for months or years (as in Burkina Faso between 2012 and 2018, in Niger between 2011 and 2017 or Zimbabwe between 2014 and 2018)

### Economic and political conditions

Complaints about inadequate remuneration and delayed payments, were the most common causal factor cited (90% of events; 63/70), followed by protest against the slow implementation of a previously reached agreement, or against the health sector’s governance and policies (36%; 25/70). Complaints about working conditions and security issues were mentioned in 14% (10/70) of the events.

Strike episodes were reported during years of weak as well as strong GDP growth, with a median growth of 4.51% (standard deviation, SD: 1.96) and an unemployment rate of 5.12% (SD: 2.80) in the affected countries (Table 2). Although strike episodes appeared to be more frequent in more recent years, no specific variable was identified for this pattern. We found little quantitative information on salary differentials between the public and private sector, but in several cases salary levels for other public servants were reported to be a reference in the negotiations (such as for physicians and senior levels of the judiciary for Mozambique in 2013, and for junior and specialist doctors in Niger 2017).

**Table 2:** Strikes episodes in 23 low-income countries and duration per year, average GDP growth and unemployment, 2009–2018

Year	No. of strike episodes	Average GDP per capita, US$	Duration, days	GDP growth, average %	Unemployment, average %^a^
2009	3	598	105	2.06	2.39
2010	2	409	40	2.82	4.06
2011	3	542	9	5.09	2.83
2012	5	640	94	4.56	2.65
2013	8	629	61	7.85	11.88
2014	10	769	31	4.68	4.61
2015	5	592	10	4.09	4.14
2016	7	717	190	2.65	6.10
2017	10	702	165	4.26	4.66
2018	17	884	170	4.40	4.13
**Total**	**70**	**717**	**875**	**4.51**	**5.12**

GDP: gross domestic product: US$: United States dollars.

^a^ Labour market statistics for low-income countries often lack accuracy and consistency,^27^ and therefore should be interpreted with caution.

Note: Table 1 lists the countries included in this table.

Data sources: The World Bank,^19^ International Monetary Fund^23^ and International Labour Organization.^24^

### Actors involved

We identified 62 reports containing information about stakeholder involvement, including professional trade unions (general and health sector specific), medical and clinical associations and government authorities in charge of negotiations (health ministry, finance ministry, President, Prime Minister or Cabinet). Striking parties were represented by professional associations, and by diverse government institutions, such as the health ministry, Presidency, Prime Minister Office and the finance ministry. Health professional councils and associations, rather than general trade unions, were involved in all the strikes identified.

Industrial action involving more than one professional category was the most common strike modality (46%; 32/70 of strike events reported), followed by strikes by physicians only (31%; 22/70 of strike events reported). Only in Zimbabwe in 2018 we found reports of nurses striking independently from other health professionals.

Reports of violent confrontation with the government were found in four cases. No explicit mention of specific mechanisms of dispute resolution was found in the reports.

Resolution was more frequently reached when other ministries (finance or public administration ministry) or higher levels of decision-making (such as Prime Minister or President) were involved, rather than the health ministry alone. According to the reports, external international actors were rarely involved in the negotiations, with the notable exception of human rights nongovernmental organizations (NGOs) in the United Republic of Tanzania in 2012 and Chad in 2018, and the World Bank’s intervention in Guinea Bissau’s health and education workers’ strike.^28^

## Discussion

This study analyses health workers’ strikes in low-income countries and links the phenomenon to a theoretical framework. Future studies will be able to build on this baseline study and use it for monitoring trends. As we mostly extracted information from online media and press reports, the study provides some unknown level of comprehensiveness. Volumes of internet users and reports from low-income settings have evolved unevenly in recent years and therefore our searches might have missed information from countries with lower access to internet services.

Although our findings are not fully comparable to the OECD data on work days lost to strikes per thousand workers in high-income labour markets,^22^ our results show that in low-income countries health workers’ strikes have become more frequent in recent years. However, the consequence for the patients, due to the disruption of health-care provision for a substantial number of days over the decade, is unknown. Understanding and monitoring heath workers’ strikes is therefore important, as such events could slow down the progress of achieving UHC. 

We were not able to find reports of health workers’ strikes for eight low-income countries during the years 2009 to 2018. This could be due to several factors. First, information may not have been readily available on the internet for these countries. Second, a substantial portion of health workers have been employed by international NGOs in these countries, making public sector industrial action less noticeable. Third, public health workers may also be engaged in private provision of services, therefore reducing the impetus of strikes.^29^ Finally, in some countries strikes in the health sector are simply not permitted.^30^^,^^31^

Although wage demands were central to most of the strike events reviewed, macroeconomic conditions, such as GDP growth, unemployment and absolute salary levels, did not appear to be key triggers. Relative pay gaps between junior and senior cadres or with other professions were mentioned as a more frequent source of recrimination..^32^^,^^33^

Our data were not sufficient to allow the identification of specific polictical economy factors for the strike episodes; however, our results do suggest that professional associations, government departments, health sector and labour market governance, all contribute in reaching positive resolutions. In physician’ cases, as senior doctors have traditionally been well-connected with the government, they have had more effective means of influencing governments and to protect their economic interests.^34^ Therefore, strikes may arise from the failure of the medical associations to represent more junior doctors or general practitioners.^13^ To advance the understanding of health workers’ strikes, the political economy aspects of individual strikes and the implications of political actors contributing to positive resolutions need to be considered. Furthermore, investing in the development of collective bargaining systems may help reduce the scope for strikes.^22^

Our results suggest that health sector strikes are context-specific, but also share some commonalities. An appropriate research agenda should therefore encompass both case-studies of individual events and more general region-wide studies looking into wider patterns of causality. Different disciplines, including economics, sociology and political science, have so far offered isolated angles and interpretations of health sector strikes. We believe that a more integrated multidisciplinary approach would be more suitable for untangling the factors of such strikes and provide an evidence base for positive resolution of such conflicts. Better understanding of strike triggers and pathways to resolution could improve the sector’s governance, patients’ access to services, and ultimately, the achievement of UHC goals.
